# Some Essential Considerations

**Published:** 1920-06-12

**Authors:** 


### PLASTER WORK IN GENERAL PRACTICE.

### I. Some Essential Considerations.

General practitioners are often called upon to deal with cases that will be benefited by correction in plaster. To some extent they are taught the advantages of plaster in their student days. All of us have learned how to make a plaster splint for a fractured tibia, how to apply it, and how to deal with it afterwards. But even in hospitals the proper artistic technique of plaster work is not carefully taught. simply because the surgeons have never learned it themselves. When one has experienced the discomforts and inconveniences of a plaster splint applied by some one who is not wholly familiar with the niceties of the work, one is in a position fully to appreciate the importance of attention to details that are now very often overlooked.

### Less Well-known Features.

By way of preface it may be mentioned that plaster work should not prove beyond the scope or ability of the medical man who has been taught the elements of his art. Men who are more handy, who have an aptitude for handicraft, will of course produce more "showy" work with plaster as they will produce more showy work with wood or with plastic operations of any kind. But between efficient work and efficient work plus artistic appearance and finished get-up there is only the difference of showiness; the quality of the work should be the same so far as its efficiency for surgical purposes is concerned. One man may fashion a splint with the utmost neatness; another will manufacture an equally serviceable and comfortable splint that will seem rough and unskilfully framed to those who are not acquainted with plaster work. But so long as the quality of the second attempt, so far as purposes of treatment are concerned, does not fall below that of the first, the second may be called good plaster work. Roughness and unskilled handiwork, in an artistic sense, will not invalidate the usefulness of the completed article; inattention to essential details of construction, on the other hand, will.

Nor is it necessary that the practitioner who wishes to work with plaster should have a large armamentarium or a fully equipped workshop. Of course, if he has the necessary instruments for finishing splints, for combining with his supporting plaster iron, brass, steel, and aluminium strengtheners, for trimming these again with leather, felt, and soft flannel, his finished article will look far superior to that of the man who contents himself with such aids as he has at hand. But here again, finish is not everything, and equally efficient plaster work can be executed with the help of such materials as are found in every household.

The use of plaster in surgery is by no means modern. As a support clay or cement of some sort was used by the old Egyptian practitioners in times that are almost prehistoric. In mediæval times it was again used principally as support and as a binding material, but its use was by no means general, and was in reality confined to a few practitioners. It found no general favour until the beginning of the last century, when its suitability as a strengthening medium was recognised by military surgeons. Baron Larrey, on his famous campaign in Russia, used clay with which cement or lime was incorporated, for various purposes, although he does not appear to have employed it in the same way as his successors did. It was left to the surgeons who accompanied the German army in the 1870 campaign to bring the advantages of plaster as a supporting medium prominently before the profession. The first plaster splints similar to some of those we use nowadays were improvised after the battle of Gravelotte, and were found so useful and efficient that they were most favourably reported upon by the surgical staff, and at the conclusion of peace were adopted in the civil hospitals. Some of these old-fashioned military plaster splints are quaint objects; a few of them which have resisted the ravages of time, may be seen in the museums of Continental medical schools, and are interesting as showing the gradual development of the art of plaster work. Italian surgeons took up the idea and modified the technique, and a few years later French and English surgeons followed suit. So we got the old-fashioned and still very useful "Bavarian," a modification of the splint originally employed at the evacuation hospitals during the Franco-German war, and the adaptable plaster splint of which the historical model is that emanating from St. George's Hospital.

### Improvements and Modifications.

Numerous improvements and modifications of these models have been invented, and some have even been patented. Similarly, modifications in the composition have been suggested and adopted. The suggestion that celluloid might be utilised for splinting purposes was an excellent one, which we owe to an Austrian surgeon; then, later on, came rubber and its substitutes, and to-day we have some dozen substances which are used in much the same manner as plaster of Paris was originally used. But none of them, with the possible exception of celluloid, can be said to be wholly satisfactory, and certainly for general practice none is so useful as plaster by itself. Yet the practitioner who works with plaster should be familiar with them.

The application of a plaster splint or corset is a matter that demands carefulness and some study of details and even of the peculiarities of individual patients. That is a point which is very generally overlooked, yet to secure the best results it is one that should be carefully considered. Attention to these little details is well repaid after-

**Plaster Work in General Practice**—(*continued*).

wards by the greater comfort of the patient and the better results obtained with the splint. This may be called the artistic aspect of applying plaster; the artistic aspect of the technique of fashioning a splint is another matter which is of less general utility and to which we need not devote a special chapter. But preparation of the patient and of his environment is an essential preliminary that well deserves careful attention. When this preliminary is overlooked, one of two things is very likely to happen. The patient complains of inconvenience which may develop into positive discomfort if the splint does not fit or the splint is so adjusted that it becomes a positive danger, although it may not give the patient any appreciable discomfort. Thus it has happened that a splint applied for so simple a matter as the correction of a foot after an operation for tenotomy has been taken off a few days later because the foot has become gangrenous. This "surgical accident" is almost unforgivable; with ordinary care and attention to the preliminaries it should never happen in plaster work. Yet it does happen, and by no means infrequently—a proof of the fact that insufficient attention is paid to these preliminaries by both teachers and students.

